# Exploiting the Metabolism of the Gut Microbiome as a Vehicle for Targeted Drug Delivery to the Colon

**DOI:** 10.3390/ph14121211

**Published:** 2021-11-23

**Authors:** Hamid A. Bakshi, Gerry A. Quinn, Alaa A. A. Aljabali, Faruck L. Hakkim, Rabia Farzand, Mohamed M. Nasef, Naji Abuglela, Prawej Ansari, Vijay Mishra, Ángel Serrano-Aroca, Murtaza M. Tambuwala

**Affiliations:** 1 School of Pharmacy and Pharmaceutical Sciences, Institute of Biomedical Sciences, Ulster University, Coleraine BT52 1SA, UK; g.quinn@ulster.ac.uk (G.A.Q.); pr.ansari@iub.edu.bd (P.A.); 2 Department of Pharmaceutics and Pharmaceutical Technology, Faculty of Pharmacy, Yarmouk University, Irbid 566, Jordan; alaaj@yu.edu.jo; 3 The Hormel Institute, University of Minnesota, Austin, MN 559122, USA; clonehakkim@gmail.com; 4 Department of Pharmacy, School of Applied Sciences, University of Huddersfield, Queensgate, Huddersfield HD1 3DH, UK; mujiz_zi@yahoo.com (R.F.); mohamednasef103@gmail.com (M.M.N.); abuglela@yahoo.com (N.A.); 5 Department of Pharmacy, Independent University, Dhaka 1229, Bangladesh; 6 School of Pharmaceutical Sciences, Lovely Professional University, Phagwara 144411, India; vijaymishra2@gmail.com; 7 Biomaterials and Bioengineering Lab., Centro de Investigación Traslacional San Alberto Magno, Universidad Católica de Valencia, San Vicente Mártir, 46001 Valencia, Spain; angel.serrano@ucv.es

**Keywords:** colon, gut microflora, oral drug delivery, gastrointestinal technology, genetically modified bacteria, probiotics, gut metabolism, colon targeted delivery

## Abstract

The prevalence of colon-associated diseases has increased significantly over the past several decades, as evidenced by accumulated literature on conditions such as Crohn’s disease, irritable bowel syndrome, colorectal cancer, and ulcerative colitis. Developing therapeutics for these diseases is challenging due to physiological barriers of the colon, systemic side effects, and the intestinal environment. Therefore, in a search for novel methods to overcome some of these problems, researchers discovered that microbial metabolism by gut microbiotia offers a potential method for targeted drug delivery This overview highlights several drug delivery systems used to modulate the microbiota and improve colon-targeted drug delivery. This technology will be important in developing a new generation of therapies which harness the metabolism of the human gut microflora.

## 1. Introduction

The incidence of colon-associated diseases in the west has risen dramatically over the past several decades, as evidenced by conditions such as Crohn’s disease, irritable bowel syndrome, colorectal cancer, and ulcerative colitis [1]. Designing prophylactic drugs for these conditions is [2] problematic because they must cope with large pH gradients, decreased absorption, low bioavailability, and possible systemic side effects from continuous degradation [3]. In the search for novel strategies to ameliorate some of these difficulties, researchers noted that the bacterial gut microflora metabolizes host nutrients at specific locations in the colon, thereby offering a potential method of targeted drug delivery [4]. It is thought that 70% of worldwide mortality has been identified as non-communicable illnesses, including cancer, cardiovascular diseases (CVD), diabetes, chronic pulmonary disease, etc. Although traditional medicinal products (tablets, capsules, and pills) are used to treat and/or manage life-threatening and infectious illnesses, they have still demonstrated cytotoxicity, microbial resistance, and adverse response to medicines [5]. To overcome some of these unwanted consequences, research efforts have focused on efficient alternative techniques, including novel drug delivery systems, microbial delivery systems, and gene delivery systems [6,7,8]. Research has shown that various conditions such as cancer as well as cardiovascular and neurological disorders could be selectively targeted using microorganisms, i.e., bacteria, viruses, and fungi. Contrary to popular belief, these microorganisms are not always dangerous and can minimize negative consequences [9]. This is exemplified by the use of bacteria such as *Clostridum novyi* which can penetrate and inhibit the development of tumors.

The GI tract is the leading site for the action of major enzymes involved in the metabolism of food [10]. Although these enzymes can also affect the stability and availability of drugs, researchers can exploit their properties to ensure local delivery within the GI tract [11]. The intestine’s microbiome, which includes over 500 distinct bacterial species [12], is also significant for metabolism and maintaining intestinal health [13]. However, the most dominant gut microbial species that represent most of the colon flora are *Firmicutes, Bacteroidetes*, *Proteobacteria*, *Actinobacteria*, and *Fusobacteria* [14]. Most intestine microbiotas stay in the anaerobic part of the colon, where carbohydrate fermentation is the principal source of nutrition for this microbial population [15]. This knowledge has been exploited to develop non-starch polysaccharide coatings, which undergo fermentation by colonic microbiota [16,17]. 

Intriguingly, the GI tract microbiome resides not only in the large intestine but is also found in the small intestine [18]. This microbial microenvironment is believed to play a vital role in the metabolic regulation in the small intestine [19]. Contrary to the large intestine, the fate of small intestine microbiota is short due to intestinal challenges such as rapid luminal flow, fluid volume, and the secretion of bacterial compounds [19]. Besides, the volume of the small intestine microbiome composition can remarkably change over a short period and is impacted by alteration in dietary intake. Although these numbers can vary considerably, the most predominant microbes found in the small intestine are genera-specific such as *Clostridium*, *Escherichia*, and *Turicibacter*. The *Streptococcus* and *Veillonella* species are also found in the small intestine [14]. 

The influence of the microbiome of the small intestine on oral drugs, formulations, and drug absorption is still unknown. Significant pharmaceutical advances have been made to improve the local targeting of drugs in the colon. However, at present, there is limited evidence of the translational efficiency of any of this research. This could be remedied by increasing evidence-based research. This short review highlights several drug delivery systems used to modulate the microbiota and improve colon-targeted drug delivery. Much of this information will help develop a new generation of therapies harnessing the power of the human gut microflora.

### 1.1. Gut Microbiome Metabolism Specific to the Colon

Microorganisms within the intestinal tract rely mostly on undigested food for survival in the upper digestive system [20]. Most of the diet which enters the large intestines is composed of complex polysaccharides with digestive enzyme-resistant linkages. Saccharolytic bacterial fermentation usually generates helpful metabolites, whereas some bacteria use other sources of energy that generates other metabolites that are more harmful to human health [20]. Following the fermentation of carbohydrates, short fatty acids and gases are the most crucial bacterial fermentation products [21]. Generally, the gut microbiota obtains its nutrition from partially digested food. Apart from indigestible polysaccharides, the colonic microbial metabolism also offers a wide variety of complex glycans, monosaccharides, and disaccharides that are not fully absorbed on the upper GIS through excessive intake or inadequate digestion [22,23]. Many people are diagnosed with diffuse degradation inside the large intestine; except low-fermenting cellulose, undigested lignin, and complex polysaccharides are processed in the gut microbiota. Colonic organisms such as Bacteroides (resistant starch, xylan), Roseburia (resistant starch, xylan, and oligosaccharides), Ruminooccus (resistant stomach and cellulose), Bifidobacterium (oligosaccharides), Fecali-bacteria, *Enterobacteria* produce a synthesis of SCFAs (acetate, propionate, and butyrate) that is an important energy source [23]. Fermentation of the carbohydrates escape the proximal digestion and undergrowth oligosaccharides. SCFAs enhance phosphorylated protein kinase (AMPK) activity in the liver and muscle. AMPK is an essential enzyme that regulates cellular energy by boosting energy consumption and the beta-oxidation of fatty acids, and reduces fat and glycogen storage [23].

**Figure 1:**
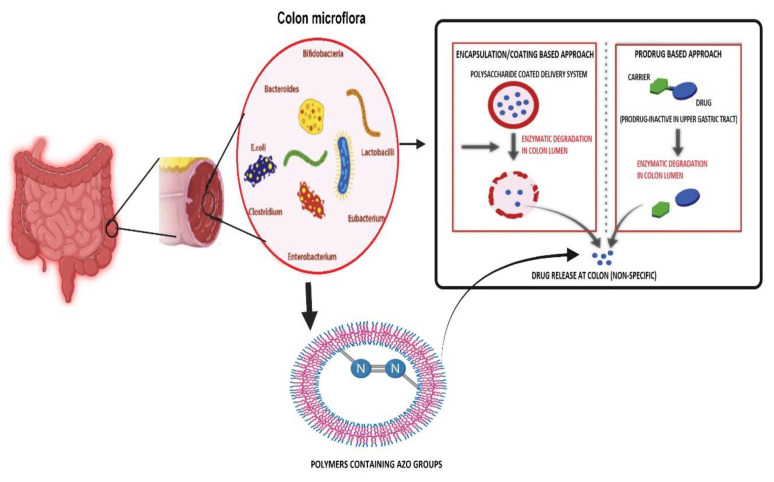
Illustrating various microbial metabolized approaches for colon target drug delivery systems. The pharmaceutical strategies that are commonly used to achieve a colon-specific drug delivery include time, pH-dependent polymer coating, prodrug, and the colonic microbiota azo group containing polymer-activated delivery systems, as well as a combination of these approaches. Image made by BioRender.

Additionally, the glucagon-like peptide (GLP) and ghrelin are essential in the glucose and energy balance through modulation of various intestinal hormone levels [24]. In addition, gut microbiota changes the peripheral fat storage by adjusting the epithelium expression of the quick-induced adipocyphal factor (FIAF), which acts as the circulatory lipoprotein lipase (LPL) inhibitor (FIAF), or the peroxisome proliferation active receptor-α (PPAR/γ) [25]. Acetate binds predominantly to the GPR43, GPR41, and GPR43 propionates and butyrate to the GPR41 for SCFAs. In the intestinal epithelium, the receptors GPR41 and GPR43 are expressed. SCFAs enhance the expression of PPARs, which are significant adipogenesis mediators. SCFAs boost leptin expression by adipocytes utilizing bindings with GPR41. Adipogenesis is supposed to be bound to GPR43. The resultant fatty acid composition might therefore be linked to obesity development [26,27,28]. Butyrate, indisputably SCFA’s most effective form of energy for human colonocytes, has a potential anti-cancer effect and can control expression levels by both inducting apoptosis in colon cancer cells and suppressing histone deacetylases [27]. Some intestinal microbes can manufacture butyrate from lactate and acetate, avoiding lactate build-up and stabilizing the intestinal environment. Propionate is a source of energy for epithelial cells and following transfer to the liver, it plays an essential part in glycogen metabolism [28]. Researchers have exploited gut bacterial metabolism to develop an array of drug delivery systems [9] based on prodrug delivery, bacterial gene therapy, polymers containing azo groups, polysaccharides containing polyol groups, and encapsulation/coating (Figure 1).

### 1.2. Prodrugs

Prodrugs are pharmaceutically inert parent drugs that are converted to active compounds in a specific environment [29]. In this instance, they are designed to bypass the harsh environment of upper GIT and can be activated in the colon by indigenous microbial enzymes [30]. The ideal prodrug requires effective distribution, metabolism, absorption, and elimination properties to be selective, safe, and stable toward the target site. Almost 10% of commercially available global therapeutics are considered prodrugs [31]. There are many different compounds that use this delivery route. However, one of the most popular delivery vehicles in recent years is polysaccharides. One example of this is cyclodextrin, a polysaccharide that resists hydrolytic stomach processes and is eventually broken down by cyclodextranase produced by bacteria in the gut [32]. Similar bacterial metabolism has been employed for glycoside/glycosidase-based prodrugs in colon-specific drug delivery. These have been used in carriers such as 5-aminosalicylic acid (5-ASA), which has been conjugated with metoclopramide (MCP) [23,33].

Other standard conjugates are routinely employed in drug trials, such as pectin coated in an enteric solution (Eudragit S100) to overcome poor compatibility [34] and chondroitin sulfate used to transport substances to the large bowel [35]. There are, of course, many other prodrugs such as amino acid conjugates, glycoside conjugates, glucuronide conjugates, cyclodextrin conjugates, and acetic acid conjugates [4]. 

### 1.3. Bacterial Gene Therapy

Specific gut microbiota, such as *Clostridium*, *Salmonella*, *Bifidobacterium*, *Listeria*, and *E. coli*, have been shown to accumulate and proliferate in the tumor microenvironment (TME). This allows for a delivery system known as bacterial-directed enzyme prodrug therapy (BDEPT) [36]. In BDEPT, patients are given genetically altered bacteria that express specific prodrug-activating enzymes and accumulate as well as secrete prodrug-converting enzymes within TME. When the enzyme levels are optimal, the prodrug is administered to patients and converted to an active drug specifically within TME [36].

Specific strains of *E. coli* DH5-lux/G have been designed to express glucuronidases that convert a sweet tasting compound usually found in liquorice root to glycrrhetnic acid in the TME. In this system, the bacteria multiply and continuously produce the therapeutic molecules that target cancer cells [37]. This BDEPT approach demonstrated that genetically engineered microbes could be an effective strategy for cancer-targeted therapy [36]. In a similar manner, recent research by a cancer group in Swansea University demonstrated that the Salmonella species could be modified to produce RNA interference, successfully reprogramming individual cancer cells to inhibit their growth. 

### 1.4. Potential of the Azo Polymer-Based Hydrogel Drug Delivery System

Another important class of colon drug delivery drugs are known as azo polymers. These are dependent on the microbial reduction of the azo bond [38,39]. Most recently, this technology has been successfully incorporated into a pH-sensitive and enzyme-sensitive nanocomposite hydrogel that delivers curcumin to colon cancer cells [40]. This demonstrated good delivery kinetics and selective targeting capacity [41]. 

### 1.5. Encapsulation

Polymers have also been used to coat drugs used in a bacterially aided drug delivery system. Polysaccharides are a common choice for this method because of their low cost, low immunogenicity, and biocompatibility [42]. Like the concept of prodrugs, encapsulated drugs are broken down by microbial enzymes at a specific location in the digestive system. However, the polysaccharide-based drug delivery system also has some drawbacks, such as high-water solubility, but further modifications can overcome this. In a similar manner, chitosan can be used as another non-toxic, biodegradable, biocompatible, and bioactive polysaccharide. In recent experiments, it has been used to produce chitosan microcores entrapped within acrylic microspheres for the colonic delivery of sodium diclofenac [43]. These can also be coated by Eudragit, ensuring efficient pH-dependent release profiles. Similarly, other polysaccharide-based drug delivery systems are listed (Table 1). 

## 2. Merits and Demerits of Colon Drug Delivery Systems

The colon specified therapeutic agents’ delivery system should be able to protect the drug enroute to the colon, i.e., drug release and uptake are not to take place inside the stomach and both the small intestine and active agent should not be degraded until it reaches the specific site in the colon [61]. For the following reasons, the colon is an appropriate absorption site for peptides and protein medications; (i) there is a much lower diversity and intensity of digestive enzymes, and (ii) the comparative proteolytic activity for the mucosa in the colon is much less than that observed in the intestines because the colon contains up to five days of extended residence and reacts quite strongly to absorption enhancements [62]. The most common and practical route for the delivery of these drugs is orally. However, alternative colon drug delivery system routes can be utilized. The quickest approach for targeting medications for the colon is rectal administration. Unfortunately, rectal administration is difficult to reach the proximal section of the colon. Rectal administration could also be discomfortable and patients’ compliance can be less than optimum [63]. For example, glucocorticoids such as dexamethasone and methylprednisolone can cause adverse effects if administered orally but administration by rectum can mitigate some of these effects [62]. Medications with limited stomach and intestinal absorption are the best agents for colon drug delivery in bowel-related diseases such as IBD, colitis, and diarrhea [64].

### 2.1. Probiotic-Aided Colon-Specific Drug Delivery

Colonic bacteria must be present in sufficient amounts at around 10 billion CFU to enable the digestion of the carrier compound (such as guar gum) and to ensure the release of colon-targeted medication. 

However, the release of drugs can be constrained by the heterogeneous nature of colonic microflora, by the sterilization of the microflora by previously administered antibiotics, and by the delays in the enzyme degradation of the carrier substances [65]. Probiotic supplements (*Bifidobacteria* spp. and *Lactobacilli*; Table 2) are reported to stimulate bacterial resistance by suppressing harmful bacterial growth, cholesterol levels, immunological response, and the production of vitamins [66].

It is well known that there is a lot of crosstalk between pathogenic bacteria and resident commensal bacteria in the gastrointestinal tract. Interruptions of these natural interactions have been related to several pathogenic diseases including ulcerative bowel disease and Crohn’s disease [76]. Therefore, some non-pathogenic live bacteria or intestinal commensals have been used to improve the host’s health and prevent or treat intestinal disorders. Lilly and Stillwell initially characterized the most useful of these organisms as “probiotic” [77]. Even though there is little clinical efficacy assessment in large and well-controlled studies, evidence is presented to support the beneficial effects of probiotics in the prevention and/or treatment of various intestinal disorders, including the recovery of pouchitis, ulcerative colitis, gastroenteritis, *Helicobacter pylori* infection, and colon cancer [78]. Recently, three cellular pathways were postulated for understanding the effects of probiotics on gut health. Probiotics firstly inhibit pathogenic bacterial effects by generating bacteriocidal chemicals and competing for gut epithelial adhesion with pathogens and toxins. Secondly, probiotics modulate immune responses by boosting innate immunity and regulating inflammation pathways caused by the pathogen. Finally, probiotics regulate intestinal epithelial homeostasis through multiple signaling pathways by stimulating intestinal epithelial cell survival, barrier function, and protection [79]. There are at least two drawbacks in the therapeutic use of probiotics: bioavailability and biosafety. In very young and immunocompromised children, for example, bacteremia linked with probiotic treatment has been described [79]. The formation of proteins generated from probiotic bacteria as new medicinal agents may be a possible strategy to tackle these issues. The discovery of pro-biosoluble substances comparable to probiotics provides insight into probiotic processes and a considerable therapeutic application [80].

The Gram-positive bacteria *Lactobacillus rhamnosus* GG (LGG; Table 2) was initially obtained from the healthy intestines of humans. LGG is one of the most researched probiotic bacteria for clinical studies to treat and/or prevent many diseases, including diarrhea and atopic dermatitis, and is frequently utilized in yogurt manufacturing as a nutritious supplement [81]. Cytokine-induced intestinal damage and apoptosis have been shown to be prevented from LGG. Furthermore, a 40-kDa protein was discovered in LGG culture, which helps protect the bowel barrier from hydrogen peroxide-induced damages by enabling the cytokine-induced apoptosis of the intestinal epithelial cells by activating the anti-apoptotic signaling of PI3K/Act [82]. Other documented impacts of LGG-derived soluble factors include promoting cytoprotective pathways in bowel cells and inhibiting the generation of cytokine in the macrophage [83].

### 2.2. Pharmaco-Microbiomes

**Figure 2:**
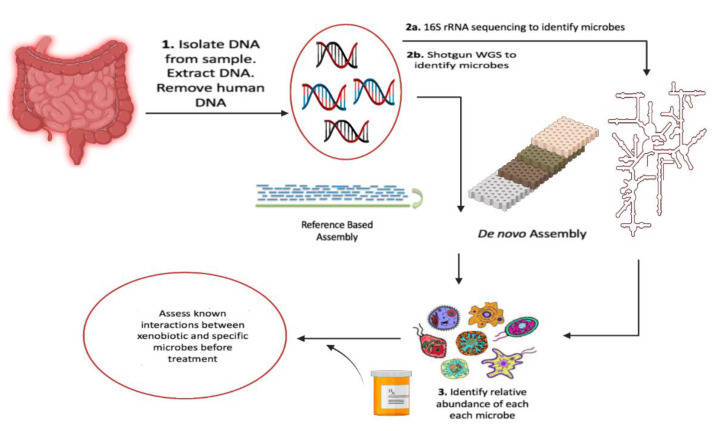
A typical pharmaco-microbiomic pipeline for the determination of appropriate xenobiotic prescriptions (image made by Biorender). A DNA from microbial samples is isolated, while rRNA is sequenced for microbes and aligned to microbial databases’ sequences. The proper xenobiotic prescriptions can be determined based on these interactions.

The human gut harbors thousands of different bacterial species and other microorganisms that form a complex ecosystem. The composition of the gut microbiome shows high inter-individual variation that is associated to several host and external factors. It can be affected by host genetics, by exogenous factors, and by their interactions. Genome-wide association studies (GWAS) have shown that the microbial composition in an individual’s gut can be affected by genetic variants involved in innate immunity, metabolism, and food processing [84]. Exogenous factors such as diet also have marked effects on the gut ecosystem: what we eat also feeds our gut microbes. A western diet and lifestyle (i.e., a high calorie, high fat diet and a sedentary lifestyle) is widely reported to be associated with a less diverse microbial ecology than, for instance, a high fiber diet [84]. Most gut microbes are strictly anaerobic and, for much of the last century, fewer than 30% of them could be cultured in the laboratory, which made functional studies impossible. With advances in culture-independent next-generation sequencing technologies, we have started to gain more insight into the composition and function of gut microbes based on their DNA sequencing [85]. Figure 2 shows a typical pharmaco-microbiome pipeline for determining the proper xenobiotic prescription, in which DNA is extracted from microbes and rRNA is sequenced, and the sequence is identified by aligning to the microbial database sequences. The microbes residing in the human gut encode a broad diversity of enzymes, greatly expanding the repertoire and capacity of the metabolic reactions in the human body that can be involved in xenobiotic metabolism, including that of dietary components and drugs. The gut microbiome is thus emerging as an important player in personalized medicine. Several papers have discussed the role of the gut microbiome on drug efficacy and toxicity [86]. 

Tremendous efforts have been made to identify human genes associated with differential drug metabolism, including an emphasis on degradation pathways. These investigations have discovered many human genetic variations implicated in the metabolism of drugs. Pharmacogenetic and pharmacogenomic projects have rendered diagnostic tests possible, supporting the selection of possible drugs (companion diagnostics). However, more than 99% of the genetic repertory of the human body accounts for the microbiome, whereas human genes contribute less than 1% [87]. Included in this wide diversity are many drug-degrading microorganisms and enzymes. Indeed, a new discipline has arisen called parmacomicrobiomics, which is involved in the understanding of mechanisms by which bacteria can degrade medicinal products [88]. Much of this information will help develop a new generation of therapies harnessing the power of the human gut microflora. Pharmacomicrobiomics have been identified as a portal resource for drug–microbiome interactions. Although more than 60 of these interactions have been identified, the underlying molecular processes and genetic bases are still mostly unknown. Some of them have been developed for specific medicines, including acetaminophen, digoxin, and cyclophosphamide [89]. The microbiota can effectively increase the prodrugs and lead to active metabolites. Sulfasalazine, for instance, is used to treat rheumatoid arthritis and cleave the azo bond that connects both active components mesalazine and sulfapyridine via bacterial azoreductase activity. However, the microbiota also reduce the bioavailability of medicines [89]. Microbial breakdown mechanisms such as digoxin may be essential [90]. The metabolites, which are processed through these pathways, might also impair the detoxifying capacity of medication. The ability to sulfonate acetaminophen to increase drug hepatotoxicity is restricted in patients with high bacterial-mediated production of p-cresol [91]. Other non-steroidal anti-inflammatory medications, such as diclofenac or indomethacin, because of the bacterial-mediated activity of β-glucuronidase, are re-absorbed into intestinal epithelial cells, leading to an increased risk of mucosal ulcers. By suppressing the bacterial activity of β-glucuronidase using the small molecule Inh-1, in vivo mucosal ulceration in animal models can be significantly decreased [92]. 

Recent emphasis has focused on the microbiome’s effect on the outcome of various cancer therapies. The immune-mediated effects of cyclophosphamide-alkylating chemotherapy disrupts the barrier function in the small intestine, allowing for Gram-positive bacteria to be translocated to the lymphatic nodes and spleen. Thus, it encourages antibiotics to reduce immune reactions of pTh17 and Th17. In a similar manenr, in a microbiome melanoma model, the absence of CpG and oxaliplatin hindered treatment responses and reduced the inherent anti-tumor reaction [93]. A similar observation was found in the mice treated with immunological checkpoint inhibitors and antibiotics, in combination improving the therapeutic’s benefits [94]. The human intestinal microbiome composition has also been found to impact the results of checkpoint inhibitors for melanoma and sarcomas therapy. Therefore, classification and immunogenicity modification of the microbiome based on the microbiome might substantially enhance the success rates for cancer immunotherapy treatments. 

The microbiome itself may be a source of novel pharmaceuticals in addition to determining the therapeutic response [95]. Recent progress in metagenomics has enabled human microbiome mining of novel biosynthesis gene clusters that generate natural chemicals which are recognized by medicinal products and isolate several small molecules of therapeutic value [96,97]. These beneficial bacteria known as probiotics have been proposed to cure diseases such as necrotizing enterocolitis, oral illness, allergy disorders, and severe depressive disorders attributed to the impact of microbiome composition in the host’s health [98]. However, the administration of a single strain in various conditions, including *C. difficile* infection, is insufficient to provide a therapeutic effect. Finally, it becomes a potential topic of study to develop microbiome commensals of diverse ranges. For example, the *E. coli* Nissle has been genetically modified to effectively lower blood phenylalanine-metabolizing enzyme levels and was assessed for the absence of phenylalanine metabolism using animal phenylketonuria models (NCT03516487) [99]. The recent advancements in cutting-edge, state-of-the-art technologies in bacterial culturomics and individualized organs-on-chips, together with the exponential growth of databanks and biobanks holding vast amounts of information about the same individual, will enable the development of the next phase in personalized medicine [100].

### 2.3. Conclusions and Future Perspectives

We have highlighted several drug delivery systems used to modulate the microbiota and improve colon-targeted drug delivery. There have also been many valuable studies on the feasibility of eliminating certain types of cancer by programming bacteria to produce therapeutic molecules or by manipulating indigenous gut bacteria to express specific prodrug-activating enzymes. However, there is limited evidence of the translational efficiency of any of this research. This is easily remedied by increasing evidence-based research. It would also be valuable to determine the consequences of the microbial metabolism of these drugs as it relates to their efficacy and toxicity. The influence of the small intestine microbiome on oral drugs, formulations, and drug absorption is still unknown. The research in this direction will open a novel window of opportunity to exploit gut enzymes and microflora for effective drug delivery in various colon-related diseases such as Crohn’s disease, irritable bowel syndrome, colorectal cancer, and ulcerative colitis.

## Figures and Tables

**Table 1:** A list of encapsulated/coated polysaccharide-based drug delivery systems for colon targeting.

Polysaccharide	Delivery System	Drug Molecule	Therapeutic Application	Feature	Ref.
Chitosan	Eudragit S-100 and chitosan-based nanoparticles	Paclitaxel	Colorectal cancer	Sustained-release, pH-responsive, bacterial enzyme sensitive, and cancer-targeted	[44]
Dextran	The doxorubicin and superparamagnetic iron oxide nanoparticles-loaded solid lipid nanoparticle coated with folate and dextran	Doxorubicin and superparamagnetic iron oxide nanoparticles	Colon cancer	The microbial enzyme sensitive and tumor-targeted delivery system used for chemo/magnetothermal combination therapy	[45]
Guar gum	The guar gum modified upconversion nanocomposite	5-Fluorouracil	Colorectal cancer	Bacterial enzyme-sensitive and NIR-triggered	[46]
Guar gum	Transformable capsules containing indomethacin immediate-release pellets	Indomethacin	Colon cancer	Bacterial enzyme-sensitive	[47]
Guar gum	Microspheres	Mesalamine and symbiotic	Ulcerative colitis	Bacterial enzyme-sensitive	[48]
Guar gum	5-Fluorouracil-containing mesoporous silica nanoparticles with guar gum capping	5-Fluorouracil	Colon cancer	Bacterial enzyme-sensitive	[49]
Pectin	The pectin/modified nano-carbon sphere nanocomposite gel films	5-Fluorouracil	Colon cancer	Bacterial enzyme-sensitive	[50]
Pectin	Pectin–zinc acetate beads coated with Eudragit S100	Pterostilbene	Colorectal cancer	pH-responsive and bacterial enzyme-sensitive	[51]
Chitosan and alginate	Thiolated chitosan/alginate composite microparticulate coated by Eudragit S-100	5-Aminosalicylic acid and curcumin	Colitis	pH-responsive, bacterial enzyme-sensitive, and mucoadhesive	[52]
Chitosan and sodium alginate	The sodium alginate-coated electrospun fiber mat containing quercetin-loaded chitosan nanoparticles and prebiotics	Quercetin and prebiotics	Colon cancer	Bacterial enzyme-sensitive	[53]
Chitosan succinate and sodium alginate	Capecitabine encapsulated chitosan succinate–sodium alginate macromolecular complex beads	Capecitabine	Colon cancer	pH-responsive, bacterial enzyme-sensitive, and mucoadhesive	[54]
Chitosan and alginate	Microcapsules	Interleukin-1 receptor antagonist	Inflammatory bowel disease	pH-responsive and bacterial enzyme-sensitive	[55]
Chitosan and pectin	Modified citrus pectinate–chitosan nanoparticles	Cetuximab and curcumin	Colon cancer	Bacterial enzyme-sensitive, mucoadhesive, and tumor-targeted	[56]
Sodium alginate and Portulaca polysaccharide	Polymeric beads encapsulating5-fluorouracil	5-Fluorouracil	Colorectal cancer	pH-responsive and bacterial enzyme-sensitive	[57]
Guar gum and pectin	Tablets coated with guar gum and Eudragit S100	Modified apple polysaccharide and mesalamine	Ulcerative colitis	Bacterial enzyme-sensitive	[58]
Hyaluronic acid and chitosan	Hyaluronic acid-coupled chitosan nanoparticles bearing oxaliplatin encapsulated in Eudragit S100-coated pellets	Oxaliplatin	Colon cancer	Bacterial enzyme-sensitive	[59,60]

**Table 2:** The effect of gut microbes in human clinical trials.

Bacterial Strain	Effects in Clinical Trials	References
*Lactobacillus reuteri*	Colonizing the intestines, primarily animal experiments thus far, perhaps a potential human probiotic	[67]
*Lactobacillus gasseri* (ADH-)	Fecal decreased enzyme and intestinal tract survival	[68]
*Lactobacillus casei Shirota*	Disease prevention, treatment of rotavirus diarrhea, balancing intestinal flora, reduction in the functioning of the fecal enzyme activities, beneficial effects on surface bladder cancer therapy, enhanced immune system in early colon cancer, and immune-boosting	[69,70]
*Lactobacillus GG* (ATCC 53013)	Preventing diarrhea linked with antibiotics, treatment, and the prevention of rotaviruses diarrhea; *Clostridium difficile* diarrhea therapy; prevention of acute diarrhea; Crohn’s disease; antagonistic against carcinogenic bacteria; vaccine adjuvant; and vaccination adjuvant	[71]
*Lactobacillus acidophilus* NCFB 1748-	Decreased colonic enzyme activity, decreased fecal mutagenicity, avoidance of diarrhea associated radiation, and constipation treatment	[72]
*Lactobacillus acidophilus* LA1	An immune-stimulating adjuvant attaching to human intestinal cells and the microflora in the intestines	[73]
*Streptococcus thermophilus*	No rotavirus diarrhea impact, no immune enhancement of rotavirus diarrhea, and no fecal enzyme activity	[74]
*Bifidobacterium bifidum*	Rotavirus diarrhea therapy, micro-flora of the intestines, and viral diarrhea treatment	[75]

## Data Availability

Data sharing is not applicable to this article.
